# Patterns of DNA Methylation in Development, Division of Labor and Hybridization in an Ant with Genetic Caste Determination

**DOI:** 10.1371/journal.pone.0042433

**Published:** 2012-08-03

**Authors:** Chris R. Smith, Navdeep S. Mutti, W. Cameron Jasper, Agni Naidu, Christopher D. Smith, Jürgen Gadau

**Affiliations:** 1 Department of Biology, Earlham College, Richmond, Indiana, United States of America; 2 Center for Social Dynamics and Complexity, School of Life Sciences, Arizona State University, Tempe, Arizona, United States of America; 3 Department of Biology, San Francisco State University, San Francisco, California, United States of America; Field Museum of Natural History, United States of America

## Abstract

**Background:**

DNA methylation is a common regulator of gene expression, including acting as a regulator of developmental events and behavioral changes in adults. Using the unique system of genetic caste determination in *Pogonomyrmex barbatus*, we were able to document changes in DNA methylation during development, and also across both ancient and contemporary hybridization events.

**Methodology/Principal Findings:**

Sodium bisulfite sequencing demonstrated *in vivo* methylation of symmetric CG dinucleotides in *P. barbatus*. We also found methylation of non-CpG sequences. This validated two bioinformatics methods for predicting gene methylation, the bias in observed to expected ratio of CpG dinucleotides and the density of CpG/TpG single nucleotide polymorphisms (SNP). Frequencies of genomic DNA methylation were determined for different developmental stages and castes using ms-AFLP assays. The genetic caste determination system (GCD) is probably the product of an ancestral hybridization event between *P. barbatus* and *P. rugosus.* Two lineages obligately co-occur within a GCD population, and queens are derived from intra-lineage matings whereas workers are produced from inter-lineage matings. Relative DNA methylation levels of queens and workers from GCD lineages (contemporary hybrids) were not significantly different until adulthood. Virgin queens had significantly higher relative levels of DNA methylation compared to workers. Worker DNA methylation did not vary among developmental stages within each lineage, but was significantly different between the currently hybridizing lineages. Finally, workers of the two genetic caste determination lineages had half as many methylated cytosines as workers from the putative parental species, which have environmental caste determination.

**Conclusions/Significance:**

These results suggest that DNA methylation may be a conserved regulatory mechanism moderating division of labor in both bees and ants. Current and historic hybridization appear to have altered genomic methylation levels suggesting a possible link between changes in overall DNA methylation and the origin and regulation of genetic caste determination in *P. barbatus*.

## Introduction

DNA methylation is a common form of epigenetic modification present in most kingdoms of life. DNA methylation can act as a major regulator of gene expression, in some contexts regulating multiple canalized phenotypes and thus giving rise to developmental and behavioral variation [1]–[6]. In some cases the DNA methylation patterns are remarkably plastic such that environmental stimuli trigger changes in DNA methylation, thus regulating transcription (reviewed in [7], [8]), and/or potentially altering patterns of gene splicing [9], [10]. Patterns of DNA methylation can also be inherited from one generation to another thereby allowing epigenetic marking of the genome to be stably transmitted through multiple cell divisions [11], [12].

DNA methylation studies in the honeybee have highlighted its potential as a major regulator of quintessential social processes, such as phenotypic plasticity and division of labor. Studies have documented a higher level of CpG methylation in the honeybee relative to solitary insects [13], [14], high levels of CpG methylation seem to be common across multiple origins of eusociality in the Hymenoptera [15], and differential DNA methylation during both developmental (larval) and behavioral (adult) division of labor in honeybees [16], [17]. Furthermore, the queen phenotype in honeybees can be produced via RNA interference knockdown of the enzyme responsible for *de novo* DNA methylation, DNMT3 [18]. This suggests that decreased *de novo* methylation during larval development will initiate queen development.

The recent sequencing of seven ant genomes, coupled with bioinformatic predictions and multiple assays to experimentally determine methylation, have recently confirmed active methylation in four of the sequenced ant species [19]–[23]. These studies have also found that genes involved in larval development leading to phenotypic plasticity have higher predicted levels of methylation compared to the genomic background [20]–[25]. Because DNA methylation can be remarkably dynamic, with environmental stimuli triggering changes in methylation, and consecutively, changes in gene expression [7], [8], DNA methylation is a likely candidate to regulate phenotypic plasticity in ants.

**Figure 1:**
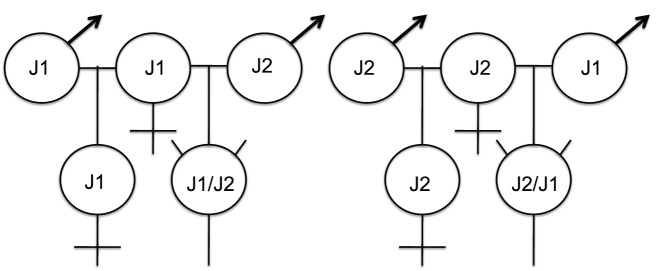
In the dependent lineage (genetic caste determining) *Pogonomyrmex*, queens obligately mate within and between lineages (the lineage pair J1/J2 are pictured here) in order to produce a functional colony. Hybrid matings produce workers (horned symbols), while within lineage, “pure”, matings produce reproductive females (future queens). Diagnostic microsatellite markers can be used to assess the parentage of individuals at any point during development, even prior to their physical differentiation.

Some lineages of the harvester ant *Pogonomyrmex barbatus/rugosus* complex have genetic caste determination [26]–[28], while in most other social insects, female caste (worker and queen) is determined by environmental differences such as the quality and quantity of larval diet [29]–[32]. Populations of harvester ants with genetic caste determination require two “dependent” lineages [29]. Both lineages must co-occur and mate in order to produce viable and reproducing colonies (Fig. 1) because offspring derived from within-lineage matings generate queens while offspring from between-lineage matings develop into workers (Fig. 1) [33], [34]. Queens in these populations are obligately polyandrous and mate with males of both lineages, but because workers are functionally sterile, there is very little gene flow between the lineages [35], [36]. This type of genetic caste determination allows us to determine the caste of an individual as soon as the queen lays an egg by using well-established genetic markers.

Using the J1–J2 dependent lineages of *P. barbatus* [37], [38], our first objective was to experimentally confirm DNA methylation in *P. barbatus* using sodium bisulfite sequencing, and evaluate the effectiveness of two methods of predicting *in vivo* methylation: bias in CpG dinucleotide values and high densities of CG/TG single nucleotide polymorphisms (SNPs) [14], [20], [25], [39], [40]. Second, we sought to determine whether gynes (i.e., virgin queens) and workers differed consistently during multiple developmental stages (larvae, pupae, adult) in overall DNA methylation. A difference in DNA methylation during early ontogeny would be consistent with data from the honeybee [18] and would suggest a role of DNA methylation in caste determination in *P. barbatus*. Third, because all workers are hybrids from inter-lineage matings, and thus genetically similar, we reasoned that a methylation difference between workers of different combinations of parents (i.e., the lineage of the mother and father) would be evidence for either variation in methylation between lineages or the result of inter-lineage hybridization.

## Results

Larvae, pupae, and adults were sampled primarily from 14 lab colonies of *P. barbatus* from a GCD population (Lat. 31.9237, Long. −109.0877), nine of which were overwintered to stimulate gyne production [41]. Overall, 235 individuals were sampled representing all developmental stages and female castes. All individuals were assigned a caste fate (gyne or worker) based on their genotypes at three microsatellite loci diagnostic for the J1 and J2 lineages [38].

### Sodium Bisulfite Sequencing

We generated site-specific methylation datasets from bisulfite treated samples for three genes in order to experimentally verify the occurrence of DNA methylation in *P. barbatus*. Genes with a higher than expected density of CpGs are known to have less methylation in other species [14], [39], [40], and the opposite is true for genes with a lower than expected density of CpGs; this pattern arises because methylated cytosines are more prone to spontaneous deamination and conversion to thymine. By the same logic, a high number of CpG<->TpG SNPs should also be suggestive of methylation. To verify these predictive methods in *P. barbatus* we chose one gene with a higher than expected CpG density (high CpG[o/e]), *egr-1*, one with a lower than expected CpG density (low CpG[o/e]), *zuc-1*, and a gene with a high density of CpG<->TpG SNPs, *fpps*. The conversion efficiency for our protocol was tested by bisulfite treatment of PCR amplified fragments of the same genomic regions for the *egr-1* and *zuc-1* genes. Of the 536 total cytosines in these control reads 526 were converted (98.1% conversion). Conversion efficiency was even greater when only considering CpG sites (155/156 = 99.3%). These controls suggest that false positives due to incomplete conversion occur at a frequency of approximately 1%.

**Figure 2:**
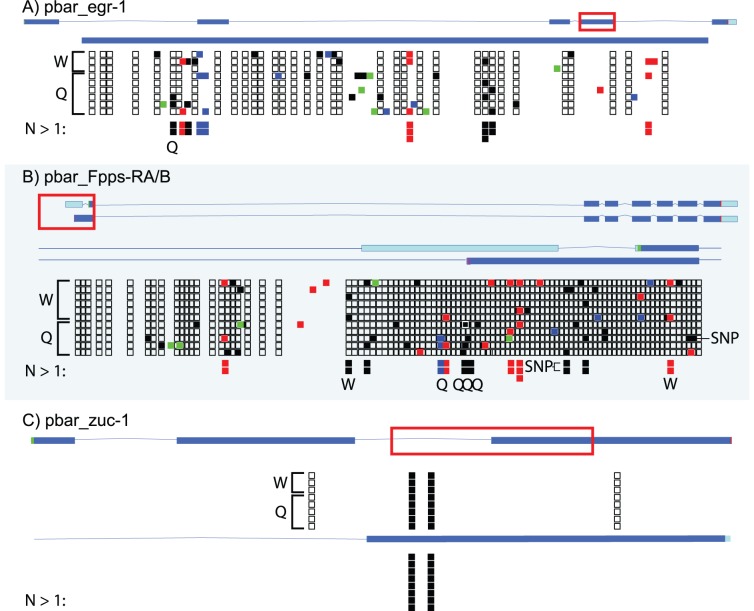
Methylation profile for three genes. The red rectangle represents the portion of this gene that was amplified by the nested primers. Below the full gene figure is a zoom view of the amplified gene area. Below the amplified area are squares representing the methylation profile of the area. Open squares represent an unmethylated CpG. Black squares represent a methylated CpG. Red squares represent a methylated CpA, Blue squares represent a methlyated CpC, Green squares represent a Methylated CpT. “W” stands for “worker”. “Q” stands for “queen”. Below each methylation profile is an “N>1” sequence which displays sites found to be methylated in more than one sample. The number of squares represents the number of times the site was found. A “Q” or a “W” was placed under the site denoting if the multiple-sample site was relegated to that caste.

Of the 28 samples from six workers and eight gynes that returned sequence from bisulfite treated genomic DNA (Table 1), we recorded 138 instances of *in vivo* methylation on 99 total sites (Fig. 2). For the genes tested, approximately 3% of all cytosines were methylated (CpN), and 10% of cytosines in CpG dinucleotide combinations were methylated. The proportion of converted cytosines after bisulfite conversion were significantly different in samples with bisulfite treatment prior to PCR (experimental) and those after PCR (control, since PCR products are not methylated) (χ2 test: P = 0.0044), strongly suggesting that our documentation of methylation is likely biologically real and not a result of false positives. Similarly, the proportion of converted cytosines in CpG dinucleotides in experimental versus control samples was highly significant (Fisher exact test: P = 0.0001). However, despite finding evidence of numerous non-CpG methylation, the difference between experimental and control samples was not significant (Fisher exact test: P>0.05), though some of these were consistent across samples (see Fig. 2 and below) suggesting that they are not spurious false positives. The majority (n = 54, ∼55%) of sites were of CpG methylation. The majority of non-CpG evidence was that of CpA methylation (n = 21, ∼23% of total methylation) but there were some examples of both CpC (n = 13, ∼21%) and CpT (n = 11, ∼11%) methylation. For the genes studied, there was no significant difference between gyne and worker averages of all methylated sites (8.375 sites/queen vs 8.000 sites/worker, t-test,: P>0.05). Overall, 23 sites had evidence for methylation across multiple samples, 13 CpG, 7 CpA, and 3 CpC (none were CpT). The low CpG[o/e] gene (*zuc-1*) exclusively featured the same two methylated CpG sites in all samples (Fig. 2). In contrast, the high CpG[o/e] gene (*egr-1*) and the high SNP gene (*fpps*), both had very few repeated sites among samples (Fig. 2), but had more overall instances of methylation. In addition, all examples of non-CpG methylation were found in the *egr-1* and *fpps* genes, while none were detected in the *zuc-1* gene.

**Table 1:** The frequency, and mean, of each type of CpN methylation observed for each gene studied in all samples.

	*egr-1*				*zuc1*				*fpps*			
	PB_12707				PB_16156				PB_23498 [2]			
	CG	CA	CT	CC	CG	CA	CT	CC	CG	CA	CT	CC
Worker #1	4	2	0	2	2	0	0	0	5	8	1	1
Worker #2	5	3	0	0	2	0	0	0	4	2	0	0
Worker #4	ND	ND	ND	ND	ND	ND	ND	ND	1	1	0	0
Worker #5	0	0	1	0	2	0	0	0	1	0	0	0
Worker #6	ND	ND	ND	ND	ND	ND	ND	ND	2	1	0	0
Worker #8	ND	ND	ND	ND	ND	ND	ND	ND	2	2	0	2
Worker Control	1	0	5	1	0	0	0	0	ND	ND	ND	ND
Queen #1	ND	ND	ND	ND	2	0	0	0	8	2	1	0
Queen #2	4	1	1	3	2	0	0	0	1	1	0	1
Queen #3	1	0	0	0	ND	ND	ND	ND	ND	ND	ND	ND
Queen #4	1	1	1	0	2	0	0	0	8	1	1	2
Queen #5	4	0	0	1	2	0	0	0	5	2	2	1
Queen #6	ND	ND	ND	ND	ND	ND	ND	ND	4	2	0	0
Queen #7	3	0	1	1	2	0	0	0	ND	ND	ND	ND
Queen #8	1	2	2	2	ND	ND	ND	ND	ND	ND	ND	ND
Queen Control	0	1	2	0	0	0	0	0	ND	ND	ND	ND
AVG Worker	3.0	1.7	0.3	0.8	2	0	0	0	2.5	2.3	0.2	0.5
AVG Queen	2.3	0.7	0.8	1.2	2	0	0	0	5.2	1.6	0.8	0.8

CpG<->TpG SNPs were discovered both within the raw reads of the published genome [18] and between our fragment sequences and the published genome. Notably, we found 12 instances of a methylated cytosine at a location the reference sequence indicated to be thymine. We found two methylated sites of the 13 reference sequence SNPs within the *egr-1* gene in two separate samples. These data suggest that the frequency of CpG<->TpG SNPs is a reliable predictor of methylation and may provide an additional computational tool (in addition to CpG[o/e]) for *in silico* prediction of methylation.

### Methylation Differences during Development

A methylation-sensitive amplified fragment length polymorphism (ms-AFLP) assay was used to estimate differences in the genome-wide DNA methylation frequency among different developmental stages of workers and gynes, workers from different lineages and workers from ECD and GCD populations. On average, 76 bands were analyzed per individual, 11 bands per primer pair (the minimum average number of bands analyzed for any group, e.g., worker larvae, was 50). Scoring of AFLP gels was done blindly with respect to sample identity. A locus was considered methylated if: 1) there was clear evidence of amplification, and 2) there was a band present for only one of the restriction enzymes (that is, MspI cut but HpaII did not, or MspI did not cut but HpaII did). To verify that scoring of AFLP gels, and the resulting patterns of methylation, were consistent across primer pairs we constructed a correlation matrix of the proportion of methylated loci per primer pair. If consistent we expected positive correlations across the primer pairs, a lack of correlation across primer pairs would suggest that one or multiple primer pairs are biasing the results (and suggest a systematic/methodological bias). All correlations between primer pairs were positive and 68% were statistically significant indicating that the patterns we present are general across all primer pairs and lack inherent bias, and thus represent relative methylation differences among individuals. It is noteworthy that the levels of methylation we report (i.e., the proportion of examined loci that are methylated) are higher than other studies on social insects done using the same methods [15]. This is likely the result of the number and visibility of bands because we also detected/examined fewer bands compared to a previous study [15]. Therefore, levels of methylation should be interpreted very cautiously across studies due to differences in gel scoring. We did not combine data for a given locus across gels because of difficulties in matching up locus lengths.

**Figure 3:**
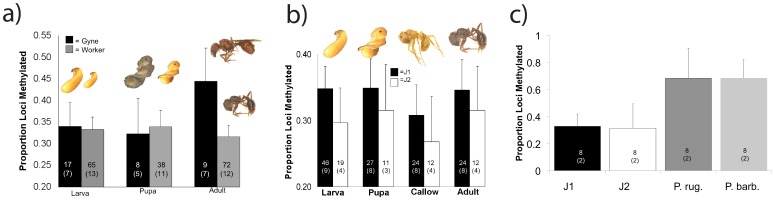
Global trends of CpG methylation. a) The proportion of methylated loci increases in queens in adulthood, but is constant for workers over development. Only adult workers and virgin queens differed, with virgin queens having significantly more methylated DNA (P<0.05). In b) variation in the proportion of methylated loci between workers of different hybrid origin. Labels indicate the maternal lineage; all workers are hybrids between the J1 and J2 lineages. The direction of hybridization affected the degree of methylation, with workers from J1 mothers and J2 fathers being 17% more methylated (P<0.05) than those from the reciprocal cross. In c) a comparison between four lineages: *P. barbatus* and *P. rugosus* have normal (environmental) caste determination and ancestrally hybridized to give rise to the J lineages (which have genetic caste determination). The J lineages are significantly less methylated (P<0.05) than their parental species. Together, b) and c) show that two successive rounds of hybridization both changed the degree of genome methylation present in workers. All error bars are 95% C.I. Numbers inside the bars indicate sample sizes, and the number of colonies from which individuals were sampled (in parentheses).

We did not detect an overall difference in the proportion of methylated loci (e.g., loci where samples differed in band presence depending on whether they were treated with MspI or HpaII) between castes (F_1,203_ = 2.95, P = 0.09) or developmental stages (F_2,203_ = 1.76, P = 0.17), but the interaction was significant (F_2,203_ = 3.62, P = 0.03) such that the gynes (adult virgin queens) were characterized by higher DNA methylation levels (Fig. 3a). In a *post hoc* comparison, gynes had significantly more methylated loci than adult workers (P = 0.02), but none of the other groups differed significantly. Workers from both J1 and J2 colonies, but only J1 gynes were included in this analysis. None of the J2 colonies sampled produced gynes in this study, although they were treated in the same way as the J1 colonies. We replicated the above analysis using only individuals from J1 colonies and the results were consistent with those reported above.

### Methylation Differences with Current and Ancient Hybridization

We tested for differences in methylation between the maternal/paternal lineage of workers, all of which are hybrids of the J1 and J2 lineages, also using ms-AFLP. Workers produced by queens from the J1 lineage had approximately 17% more methylated loci than workers produced by J2 queens (F_1,167_ = 4.63, P = 0.048), and this difference was consistent across all developmental stages (developmental stage × lineage: F_3,167_ = 0.09, P = 0.98) (Fig. 3b). Additional samples were used to assess how the methylation levels observed above corresponded to the parental species of the J1 and J2 lineages. The genomes of both genetic caste determining J-lineages have hybrid character, the result of ancient hybridization between *P. barbatus* and *P. rugosus,* which have normal environmental caste determination (ECD). The genome structure of the J lineages reflects an ancient hybridization between ECD *P. barbatus* and *P. rugosus*, [38], [42]. Hence we tested whether 1) the methylation patterns in the J1/J2 lineages are derived, possibly as a result of genetic caste determination, and 2) whether the higher levels of methylation observed in lineage J1 may be elevated over the levels of the parental species because lineage J1 has a greater hybrid character (see discussion). We sampled four workers from two colonies of each J1, J2, ECD *P. barbatus*, and ECD *P. rugosus*. Workers of both ECD species had much elevated (double) levels of methylation relative to J1 and J2 (Fig. 3c). In *post hoc* comparisons, the ECD colonies did not differ from each other, the J1 and J2 colonies did not differ from each other, but all ECD workers had significantly higher methylation levels than all J1/J2 (P<0.001).

## Discussion

Our results corroborate active DNA methylation in ants and for the first time demonstrate that differences in methylation levels are correlated with development and division of labor in ants. The presence of an active methylation system in ants was demonstrated in previous studies [15], [19]–[21], and is confirmed here for *P. barbatus* using two complementary methods, gene-targeted sodium bisulfite sequencing and methylation sensitive AFLP (ms-AFLP) analysis. Our bisulfite sequencing experiments detected cytosine methylation in both adult workers and virgin queens (gynes). As expected based on theoretical predictions [39], genes with low CpG[o/e] (i.e., a lower than expected density of CpG dinucleotides) showed fewer but the most consistent site-specific methylation (Fig. 2). Regions with low CpG[o/e] ratios are suggestive of long-term germ-line methylation [14], [43]. Conversely, as honeybee (*Apis mellifera*) genes associated with caste determination were found to contain higher CpG[o/e] values [14], such genes in *P. barbatus* may be more evolutionarily recent areas of methylation activity, such that the methylated sites have not yet been fixed over time. Using ms-AFLP we detected no consistent difference between castes or developmental stages in genome wide methylation, but did find that adult gynes had higher methylation levels than adult workers. Furthermore, we found a significant difference in methylation levels between workers of each maternal lineage (all workers are hybrids), and a difference in methylation among workers from genetic caste determining (GCD) lineages and the environmental caste determining lineages from which they likely evolved (via ancient hybridization) [35]. These findings suggest that genome wide methylation patterns have been altered by past and current hybridization, and that evolving patterns of methylation could play a role in the evolution of genetic caste determination.

### Gene Specific *in vivo* Methylation

We have demonstrated the validity of using CpG[o/e] values and SNPs to predict the presence of methylated cytosines in a specific region of DNA in *P. barbatus*. Here we measured the levels of DNA methylation in 3 genes in adults (gynes and worker samples) by sodium bisulphate sequencing and found that approximately 3% of all cytosines and 10% of all cytosines in CpG dinucleotides were methylated, and 22% of methylated sites were found repeated across at least two samples (n = 23 sites). We observed two patterns of methylation: hyper-methylated genes with low CpG[o/e] where methylated sites were conserved across all samples (*zuc-1*), and hypo-methylated genes with high CpG[o/e] where methylated sites were located at variable positions across the region among samples (*fpps*, *egr-1*), but had much higher raw numbers of overall methylation (Fig. 2, Table 1). The higher actual levels of methylation in the higher CpG[o/e] gene supports that many genes with low predicted levels of methylation may actually be targets of *de novo* methylation rather than germ line methylation, and that CpG[o/e] values alone may miss many methylated genes [40].

We expected gynes to be transcriptionally more inactive because they are essentially quiescent, waiting to leave the nest for nuptial flights. Thus we predicted that they would have higher amounts of methylation, silencing gene networks responsible for reproduction and labor. For the *fpps* gene, we observed slightly higher, though not significant, methylation in gynes by bisulfite treatment (Table 1) and methylation sensitive analysis. However, our analysis focused on three genes and the ms-AFLP was genome-wide. Comparing methylome landscapes between queens and workers would determine what genes and pathways are affected by methylation and perhaps regulate adult reproductive division of labor.

It is notable that we detected non-CpG methylation at higher than expected rates (Table 1). Other studies [44] have found infrequent instances of CpC methylation but have explained it as possible result of incomplete bisulfite conversion. Incomplete conversion may explain the non-CpG methylation here as well although it is notable that conversion efficiency was high (98.1%) and we noticed the same non-CpG sites methylated in multiple samples in some cases (Fig. 2). CpA and CpT methylation have been demonstrated in stick insects [44] and three of the examples of CpC methylation in our results were found at the same site in multiple samples. At least in humans, non-CpG methylation is positively correlated with DNMT3 activity and CpG methylation [45]. In agreement with this, non-CpG methylation was only observed in two of the three genes assayed in our study, and those two genes showed the highest levels of methylation (*egr-1* and *fpps*). Due to its correlation with DNMT3 activity, it has been proposed that non-CpG methylation may be stochastic, though biological hypotheses for non-CpG methylation have also been proposed [45].

### Caste Differences in Methylation Across Ontogeny

Genomic DNA methylation was similar between gyne and worker castes in the larval and pupal stages, but increased in adult gynes relative to adult workers (Fig. 3a). The increase in gynes could serve a regulatory role. Genes with caste-specific expression, at least in honeybees, tend to have higher than expected CpG density and may be regulated by methylation (though would tend not to be germ-line methylated) [14]; furthermore, these same genes tend to evolve at a rapid rate relative to non-caste specific genes, potentially facilitating novel functions [46]. After eclosion, but prior to dispersal, gynes develop oocytes [47] and increase protein and carbohydrate energy stores [48]. Increased methylation may regulate the genes/networks controlling this quiescent phase, which is completely absent in adult workers. Compared to adult gynes, adult workers engage in many tasks and this difference in behavior may be in part regulated via *de novo* CpG methylation of genes/networks.

Our data are insufficient to test for differential methylation during queen-worker differentiation in larval development, as was previously demonstrated in the honeybee [18] because larvae of all stages were pooled for analysis. Knowledge is currently lacking on when during development the castes begin to differentiate in *P. barbatus*, but when this point is found it will be informative to assay DNA methylation levels and observe how genes and pathways might be modified by methylation of cytosine’s in order to orchestrate female caste differentiation. While our results cannot support a convergence in methylation in caste determination between the evolutionarily independent eusocial lineages of honeybees (*Apis*) and ants (*Pogonomyrmex*), they are consistent with DNA methylation as a mechanism for in generating caste differences, at least between adult workers and gynes.

### Differential Methylation with Hybridization

The genetic caste determining lineages of *Pogonomyrmex* are a genomic mixture of the parental lineages *P. barbatus* and *P. rugosus* with environmental caste determination and hence are derived from an ancestral hybridization event [29]. Based on a combination of mitochondrial sequencing and nuclear microsatellite genotyping, J1 has a 75% *P. rugosus*/25% *P. barbatus* nuclear genome and a *P. rugosus* mitotype, while J2 has a 1% *P. rugosus*/99% *P. barbatus* nuclear genome and a *P. barbatus* mitotype [38], [42]. Our data show that DNA methylation levels are significantly decreased in the J lineages compared to the parental species (Fig. 3c). Furthermore, the J lineages obligately hybridize to produce workers, and our data show a difference in worker methylation dependent on the direction of the hybrid cross. The DNA of workers born from J1 mothers and J2 fathers has significantly higher methylation levels than the reciprocal cross, and these levels are maintained across all developmental stages (Fig. 3b). Studies on both animals and plants have shown changes in the extent of DNA methylation resulting from hybridization [49]–[51] and our data extend these observations to insects. The decrease in methylation from the parental *P. barbatus* and *P. rugosus* to the J lineages is approximately half, possibly the result of differential methylation of genes in each of the parental species resulting in only one allele being methylated in the hybrid. Differences in the level of DNA methylation based on the direction of hybridization between the J lineages is suggestive of inherited differences in methylation such that DNA in the germ line is differentially methylated in each lineage. Of note, the levels of CpG methylation reported in the parental *P. barbatus* and *P. rugosus* are very high (Fig. 3). The global CpG methylation levels reported from ms-AFLP is generally much higher than we report from bisulfite sequencing. It is likely that the ms-AFLP method over-estimates methylation, but even so, an interesting next step is to investigate the evolution of methylation in these lineages, especially as it corresponds to changes in the regulation of development and division of labor.

### Conclusions

We demonstrate using, multiple methods, that DNA methylation occurs in the red harvester ant, *P. barbatus*. Cytosines are not only methylated in CpG dinucleotides, but in all dinucleotide combinations. We also show that CpG methylation levels vary between adult gynes and workers, suggesting a role of DNA methylation in behavioral division of labor. Lastly, the ant we studied originated from ancient hybridization, and currently must hybridize to form viable colonies, and we show that methylation levels differ between the proposed parental lineages and their hybrids, and that when the hybrid lineages again interbreed (to form colonies) that the level of methylation is affected by the direction of the cross. These findings open up interesting avenues of research on how methylation may help organize division of labor in ants and how methylation patterns evolve during complex hybridization events.

## Methods

### Material Collection

All J-lineage samples were from lab colonies reared from founding queens and maintained in the laboratory for two years (approximately 100–200 workers per colony). Queens were collected from a mating swarm near 31.9237, −109.0877. All of the lab colonies were morphologically determined as *P. barbatus* of the J1–J2 lineages. Colonies were kept at 30°C on a diet of water, Kentucky bluegrass seed, and one cricket per week. Nine of the colonies were “overwintered” to induce the production of reproductives [41]; larvae, pupae and adult stages were sampled after overwintering. Samples of ECD *P. barbatus* and *P. rugosus* were from mature field colonies near Querétaro, Mexico (20.6663, −100.0706) and Tempe, Arizona (32.93704, −111.69077), respectively.

### Bisulfite PCR Sequencing

Genomic DNA of six workers and eight queens was extracted using a Qiagen DNeasy Blood & Tissue Kit [Valencia, CA USA]. The DNA (300 ng/sample) was then treated with a Qiagen EpiTect Bisulfite Kit according to the manufacturer’s specifications. The bisulfite-treated DNA was then amplified with the following PCR protocol (94°C for 2 min., 94°C for 10 sec., 55°C for 20 sec., 72°C for 20 sec., repeat steps 2–4×40, 72°C for 5 min.) This protocol was then repeated using the hemi-nested primer set (Table 2). We chose three candidate genes from the genome annotation dataset [20]; one with a high predicted CpG observed/expected ratio (*egr-1*), a gene with a low predicted CpG observed/expected ratio (*zuc-1*), and one gene with a high density of CG<->TG SNPs (*fpps*) [20]. We used the Methprimer [52] web portal to design nested (or hemi-nested) primer sets (Table 2) in regions of the genes containing the highest concentration of CpG dinucleotides. Bisulfite treated samples were sequenced using the Sanger dideoxy technique by Elimbio Biosciences (Hayward, CA USA) and then compared to reference genomic DNA [20] to determine if they contained any methylated cytosine residues. Note, because all bisulfite treated sequences were compared to the genome reference to detect evidence for methylation, polymorphisms between treated and reference sequences, unrelated to bisulfite treatment, may yield some false positives. The raw chromatograms were visually inspected for any un-converted cytosine sites. Instances where sequences had thymine/cytosine mismatches relative to the reference sequence were recorded as SNPs. Modified residues were visualized in the context of gene annotations using Apollo [53] annotation tool. Control DNA sequences were generated by bisulfite-treating the genomic PCR products of genes *egr-1* and *zuc-1* and inspected unconverted cytosine residues to determine the efficiency of conversion; since the genomic PCR product is devoid of methylated cytosines then 100% of cytosines should be converted, thus providing an assay to assess conversion efficiency of the protocol. Chi squared and Fisher’s exact tests were used to determine whether the differences in cytosine conversion were statistically different between control and experimental samples.

**Table 2:** Primers used for bisulfite sequencing.

Primers
***egr-1***
Forward
TTGTTATTAAATTGTTATTAATTGTTAAGT
Reverse
AAAAATTATAAATTAAATTAAAAACAC
Forward Nested
TATTATGTATTGTAGGATAGGTTTGT
***zuc-1***
Forward
TAAAATGATATAAGTTAAATAATGAATTTT
Reverse
ACTAATTTTATCCAATTTACACTACC
Forward Nested
TTTAGAATATATTTATGATTTGAAATTATT
***fpps***
Forward
TAGAAGTTTAAAATTAAGTTTAAGTTTTT
Reverse
TCTTTAATCCATAATATAATCAACAATATA
Forward Nested
GTGTGTTTTTTTTATTTTTATTTTTAGTA

### Lineage Determination

The maternal lineage (J1 or J2) of each lab colony was determined using a mitochondrial restriction fragment length polymorphism, RFLP [33]. This protocol supplemented microsatellite genotyping and aided microsatellite gel scoring because we then knew the lineage of the queen.

The protocol we used is the same as in Anderson et al. [33]. Genomic DNA of one worker per colony (all workers contain the same mitochondrial genome as the queen) was extracted using the protocol described in [31] using 150 µl 5% Chelex. A 630 bp fragment of *cytochrome oxidase 1* was amplified using the primer pair LCO/HCO [54] with the following recipe: in a 25 µl reaction there was 15.9 µl water, 5 µl of 5X Promega GoTaq buffer (with 7.5 mM MgCl2), 0.5 µl of 50 mM MgCl2, 0.5 µl of 10 mM dNTPs, 0.5 µl of each 10 µM primer, 0.1 µl *Taq* polymerase, and 2 µl of genomic DNA. The PCR program was 95°C for 4 min, then 38 cycles of 95°C for 30 s, 45°C for 45 s, and 68°C for 90 s, and then a final elongation at 68°C for 4 min. The PCR product of LCO/HCO was digested with the restriction enzyme *SspI* using the following protocol: 15 µl of the PCR product was combined with 12.5 µl water, 2 µl *SspI* buffer, and 5 units of *SspI*, and the solution was incubated at 37°C for two hours. The digested product was examined using a 2.5% agarose gel. *SspI* cuts only in the amplified product of lineage J2; thus, if the digested product showed one band ∼600 bp in length the lineage was determined as J1, and if two bands J2.

### Microsatellite Genotyping

All individuals in this study, along with individuals from 15 field-collected colonies of known lineage (unpublished data), were screened for loci diagnostic for distinguishing the J1 and J2 lineages. Two loci, Myrt3 [55] and Pb8 [56], had no overlap in alleles between lineages, and only one allele was shared at L18 [57]. These results were in accord with previous studies [28], [38]. These three loci were used to determine whether individuals of unknown caste (larvae) were fated to develop as workers or queens.

All PCRs were carried out in a 12.5 µl reaction using 6.4 µl water, 3.5 µl of 5X Promega GoTaq buffer (with 7.5 mM MgCl2), 0.5 µl of 10 mM dNTPs, 0.5 µl of each 10 µM primer, 0.1 µl Taq polymerase, and 1 µl of genomic DNA from the above DNeasy isolation (diluted 1∶10 with water, see methods). A standard PCR program was used for each locus, 95°C for 5 min, 31 cycles of 95°C for 45 s, Ta (see below) for 45 s, 72°C for 45 s, and a final elongation at 72°C for 7 min. The locus specific annealing temperatures were: Myrt3 = 50°C, L18 = 54°C, and Pb8 = 57°C. Since larvae cannot be physically differentiated with regard to caste, and because both reproductive females and males are expected to have alleles of only one lineage (males being haploid/hemizygous), we examined loci for heterozygosity in order to be able to exclude males. No individual was homozygous for all three loci, thus confirming the absence of males from our dataset; to best of our knowledge, diploid males have not been described from *Pogonomyrmex*.

**Table 3:** Sequences of adaptors and primers used in ms-AFLP analyses.

Oligonucleotide	Sequence
*EcoR*I adaptor	CTC GTA GAC TGC GTA CC AAT TGG TAC GCA GTC TAC
*Msp*I – *Hpa*II adaptor	GAC GAT GAG TCT AGA A CGT TCT AGA CTC ATC
*EcoR*I preselect primer	GAC TGC GTA CCA ATT C
*Msp*I – *Hpa*II preselect primer	GAT GAG TCT AGA ACG GA
E1	GAC TGC GTA CCA ATT CAC
E2	GAC TGC GTA CCA ATT CAA
E3	GAC TGC GTA CCA ATT CAG
E4	GAC TGC GTA CCA ATT CAT
M1	GAT GAG TCT AGA ACG GAAT
M2	GAT GAG TCT AGA ACG GATC
M3	GAT GAG TCT AGA ACG GACT

### Methylation-sensitive AFLP Genotyping

A methylation sensitive amplified fragment length polymorphism method was used to determine total genomic methylation of sampled individuals. Approximately 500 ng of genomic DNA was extracted using a Qiagen blood and tissue kit with the supplemental insect protocol (Valencia CA) and used for two separate digestions with restriction endonucleases, one using *EcoRI* and *MspI* and the other using *EcoRI* and *HpaII*. *MspI* and *HpaII* are isoschizomers (sharing the same cutting site, CCGG), but *MspI* cuts regardless of methylation of the second C, while *HpaII* will not cut when the second cytosine in the cut site is methylated; neither will cut if the first C is methylated. Thus this technique will not document anything other than CpG methylation [58].

Five units of each enzyme were combined with 1 µl buffer, water and DNA to a 10 µl total volume and incubated for three hours at 37°C followed by 10 min at 80°C. *EcoRI* and *MspI*/*HpaII* adapters (Table 3) were ligated to the digested DNA in the following 10 µl reaction: 3 µl digested DNA, 0.05 µl of 100 uM *EcoRI* adapter, 0.5 µl of 100 uM *MspI*/*HpaII* adapter, 200 units of T4 ligase, 1 µl ligase buffer, and 5.35 µl water; this solution was incubated at 37°C for three hours and then 25°C for an additional 12 hours. Pre-select PCR was performed on the resulting blunt-ended DNA using the following 10 µl PCR recipe: 2.9 µl water, 3.5 µl 5X Promega GoTaq buffer (with 7.5 mM MgCl2), 0.5 µl 10mM dNTPs, 1 µl of each pre-select primer (Table 3) at 10 uM, 0.1 µl Taq polymerase, and 1µl of 1∶10 diluted adapter ligated DNA from above. This was amplified using the following program: 5 min at 95°C, 20 cycles of 95°C for 30 s, 56°C for 1 min, 72°C for 1min., and a final 7 min at 72°C. The pre-select PCR product was then diluted 1∶10 with water and used in eight different select PCR combinations; of the 12 possible combinations of forward and reverse primers (Table 3) we assayed 10 primer combinations and chose the eight with the most bands, these were: E1/M1, E2/M2, E2/M3, E3/M1, E3/M2, E3/M3/E4/M2, E4/M3 (E1/M2 and E2/M1 yielded little to no detectable amplification). The forward select primers (Table 3) were labeled with a 700 nm IR dye for use with the LI-COR 4300 DNA analysis system. The select PCR recipe is the same as was used for the pre-select PCR. The PCR program used for select PCR was: 5 min at 95°C followed by 36 cycles of 94°C for 30 s, annealing temp for 30 s (see below), and 1 min at 72°C, then a final elongation for 7 min at 72°C. The annealing temp in the first cycle was 65°C and then subsequently reduced each cycle by 0.7°C for the next 12 cycles, and then continued at 56°C for the remaining 23 cycles. The resulting select PCR product was diluted 1∶10 with water and then combined 1∶1 with loading dye prior to loading in a 6.5% polyacrylamide gel. The *MspI* and *HpaII* digested samples were run side-by-side on the gel to facilitate scoring. Saga^MX^ automated AFLP analysis software (LI-COR inc., Lincoln, Nebraska, USA) was used to score gels. Scoring of gels was done pseudo-blind such that the identity of the sample was known, but not its caste.

### AFLP Statistical Methods

All scoring was done blindly. Two-way ANOVA was used to test whether queens and workers differed in genomic methylation across developmental stages. In this analysis, caste (queen or worker) and developmental stage (larva, pupa, adult) were independent variables and the proportion of methylated loci was the dependent variable. Two-way ANOVA was also used to test whether the lineages differed in methylation using a sampling of workers of all developmental stages; in the model, maternal lineage (J1 or J2) and developmental stage (larva, pupa, callow adult, and pigmented adult) were independent variables and the proportion of methylated loci was the dependent variable. Reproductive females could not be included in this analysis because only J1 colonies produced reproductive females during our sampling. One-way ANOVA was used to compare methylation levels in workers collected from colonies of each lineage, J1, J2, ECD *P. barbatus*, and ECD *P. rugosus*. Where appropriate a Tukey’s HSD was used for *post hoc* comparisons.
